# The calcineurin β-like interacting protein kinase CIPK25 regulates potassium homeostasis under low oxygen in Arabidopsis

**DOI:** 10.1093/jxb/eraa004

**Published:** 2020-02-13

**Authors:** Andrea Tagliani, Anh Nguyet Tran, Giacomo Novi, Riccardo Di Mambro, Michele Pesenti, Gian Attilio Sacchi, Pierdomenico Perata, Chiara Pucciariello

**Affiliations:** 1 PlantLab, Institute of Life Sciences, Scuola Superiore Sant’Anna, Pisa, Italy; 2 nanoPlant Center @NEST, Institute of Life Sciences, Scuola Superiore Sant’Anna, Pisa, Italy; 3 Department of Biology, University of Pisa, Pisa, Italy; 4 Department of Agricultural and Environmental Science, University of Milano, Milano, Italy; 5 Michigan State University, USA

**Keywords:** Anoxia, Arabidopsis, calcineurin β-like interacting protein kinase, CIPK25, hypoxia, potassium homeostasis

## Abstract

Hypoxic conditions often arise from waterlogging and flooding, affecting several aspects of plant metabolism, including the uptake of nutrients. We identified a member of the CALCINEURIN β-LIKE INTERACTING PROTEIN KINASE (CIPK) family in Arabidopsis, *CIPK25,* which is induced in the root endodermis under low-oxygen conditions. A *cipk25* mutant exhibited higher sensitivity to anoxia in conditions of potassium limitation, suggesting that this kinase is involved in the regulation of potassium uptake. Interestingly, we found that CIPK25 interacts with AKT1, the major inward rectifying potassium channel in Arabidopsis. Under anoxic conditions, *cipk25* mutant seedlings were unable to maintain potassium concentrations at wild-type levels, suggesting that CIPK25 likely plays a role in modulating potassium homeostasis under low-oxygen conditions. In addition, *cipk25* and *akt1* mutants share similar developmental defects under waterlogging, further supporting an interplay between CIPK25 and AKT1.

## Teaser

A CIPK family protein involved in the regulation of potassium homeostasis under low oxygen was identified. CIPK25 directly interacts with AKT1, the main inward rectifying potassium channel in plants.

## Introduction

The intensification of flooding events is one of the consequences of climate change that is strongly affecting plant biodiversity and crop productivity. In a flooded environment, the availability of external oxygen (O_2_) is reduced, because gas diffusion in water is lower than in aerobic conditions (Armstrong, 1979; Colmer, 2003). Low O_2_ availability for plants is not only a consequence of environmental stress but also occurs during the development of specific organs and tissues, such as fruits, root vasculature, and seeds (Van Dongen and Licausi, 2015).

In plants, hypoxia is perceived by members of the group VII ETHYLENE RESPONSIVE FACTORS (ERF-VIIs), whose protein stability is regulated by PLANT CYSTEINE OXIDASE (PCO) enzymes in an O_2_-dependent manner (Gibbs *et al.*, 2011; Licausi *et al.*, 2011; Weits *et al.*, 2014; White *et al.*, 2017). PCO enzymes destabilize ERF-VIIs through the O_2_-dependent oxidation of an N-terminal cysteine, targeting the ERF-VIIs for proteasomal degradation. This process is prevented under O_2_ limitation, allowing ERF-VIIs to act as transcriptional activators of genes involved in anaerobic metabolism.

Together with a direct O_2_ sensing mechanism, additional signaling pathways contribute to the plant’s adaptation to low O_2_ availability. These pathways rely on perturbations of cellular homeostasis due to changes in available sugars, energy status, cytosolic calcium (Ca^2+^), pH, reactive oxygen species, reactive nitrogen species, and possibly potassium (K^+^) levels (Bailey-Serres and Chang, 2005; van Dongen and Licausi, 2015; Shahzad *et al.*, 2016; Pucciariello and Perata, 2017; Wang *et al.*, 2017*b*).

Among the second messengers, Ca^2+^ is involved in the response to many stimuli related to plant development and environmental cues (Dodd *et al.*, 2010). Release of Ca^2+^ into the cytosol from internal stores or from the extracellular space occurs under various conditions, so that different external stimuli are transduced by distinct spatio-temporal variations in the frequency, amplitude, and location of Ca^2+^ waves (Kudla *et al.*, 2018).

In line with the widespread signaling function of Ca^2+^, early reports suggested that O_2_ deprivation triggers a cytosolic Ca^2+^ flux in several plants, which indirectly regulates the expression of anaerobic genes (Subbaiah *et al.*, 1994; Sedbrook *et al.*, 1996; Nie *et al.*, 2006). Using rice protoplasts, the increased cytosolic Ca^2+^ concentrations observed under anoxia have been suggested to depend on both external and internal stores (Yemelyanov *et al.*, 2011). More recently, a CALMODULIN-LIKE PROTEIN 38 (CML38) was found to be induced under low O_2_ and associated with cytosolic stress granules in a Ca^2+^-dependent manner (Lokdarshi *et al.*, 2016).

Due to its ubiquitous role, the Ca^2+^-dependent network is multifaceted, and plants are equipped with a plethora of sensors able to transfer the message to downstream transducers. A major family of Ca^2+^ sensors is the CALCINEURIN β-LIKE PROTEIN (CBL) family, which is unique to plants. CBLs modulate the activity of CBL-INTERACTING PROTEIN KINASE (CIPK) partners, which have a catalytic activity (Weinl and Kudla, 2009), thus acting as a signaling relay in which the sensor and the effector are two separate proteins (Kudla *et al.*, 2018).

CIPKs belong to the subgroup of SUCROSE NON-FERMENTING 1 (SNF1) RELATED PROTEIN KINASE 3 (SnRK3) of plants, which is functionally similar to SNF1 in yeast and AMPK in mammals (Mao *et al.*, 2016). CIPKs have a typical structural organization consisting of an N-terminal kinase catalytic domain and a C-terminal regulatory domain (Sanyal *et al.*, 2015). The C-terminus contains the NAF/FISL motif, which is responsible for self-inhibition of the enzyme, and a protein phosphatase interaction domain. The Ca^2+^-dependent interaction of CBLs with the CIPK NAF/FISL motif activates the kinase, releasing it from autoinhibition (Chaves-Sanjuan *et al.*, 2014). Additionally, the activity of CIPKs is influenced by phosphorylation within the activation loop (Chaves-Sanjuan *et al.*, 2014).

The CBL–CIPK complex transmits the Ca^2+^-dependent signal to downstream target proteins via phosphorylation (Sanyal *et al.*, 2015). Each CBL can interact with multiple CIPKs and *vice versa*, providing a substantial level of versatility and flexibility in the Ca^2+^ signal transduction pathway (DeFalco *et al.*, 2009).

Many physiological functions have been assigned to CBL–CIPK complexes, including the regulation of ion transport, the stress response, and plant development (for a review see Kudla *et al.*, 2018). Some CBL–CIPK combinations—CBL1/9 and CIPK23 (Li *et al.*, 2006; Xu *et al.*, 2006; Cheong *et al.*, 2007), CBL4 and CIPK6 (Held *et al.*, 2011), and CBL3 and CIPK9 (Liu *et al.*, 2013)—are involved in regulating K^+^ homeostasis in Arabidopsis roots and/or modulating the activity of plasma membrane channels (Wang *et al.*, 2018).

K^+^ is the most abundant inorganic cation in plants, contributing up to 10% of their dry mass (Leigh and Wyn Jones, 1984) and having a high concentration (~100–200 mM) inside the plant cytosol (Wyn Jones and Pollard, 1983). It is crucial in several processes, such as the maintenance of cell turgor and growth, the regulation of metabolism through direct interaction with enzymes, and the regulation of ionic balance in the cell (Sharma and Sharma, 2013). In this framework, the CBL1–CBL9/CIPK23 effector module is activated under K^+^ starvation and regulates the *Shaker* inward-rectifying K^+^ channel AKT1 through interaction and phosphorylation (Li *et al.*, 2006; Xu *et al.*, 2006; Lee *et al.*, 2007). In addition, the CBL4/CIPK6 complex mediates plasma membrane targeting as well as the activity of the highly selective and weak inward-rectifying K^+^ channel AKT2 (Held *et al.*, 2011).

Under low O_2_ conditions, membrane depolarization occurs as a consequence of reduced proton pumping at the plasma membrane, due to a reduced ATP pool (Gout *et al.*, 2001). This depolarization is likely transient, since the increased concentration of H^+^ ions in the cytosol is counteracted by a rapid stimulation of depolarization-activated K^+^ efflux channels, which repolarizes the plasma membrane potential (Zeng *et al.*, 2014, Cuin *et al.*, 2018). This process may thus cause a latent K^+^ starvation. In fact, in plants exposed to low-O_2_ conditions, the K^+^ pool in the root is markedly reduced, and exogenous foliar or root applications of K^+^ alleviate the adverse effect on plants (for a review see Shabala *et al.*, 2014). In line with this finding, Arabidopsis *gork1-1* mutants lacking functional K^+^ efflux channels possess higher tolerance to hypoxia (Wang *et al.*, 2017*a*).

The modification of K^+^ flux inside the cell may also indirectly alter the fermentative metabolism activated under O_2_ shortage. Shahzad *et al.* (2016) identified a MAPKKK, HYDRAULIC CONDUCTIVITY of ROOT 1 (HCR1), which contributes to RAP2.12 (ERF-VII) stabilization under hypoxia only when K^+^ is available. Moreover, K^+^ gradients may be exploited by Arabidopsis plants as a source of energy under low O_2_ conditions, since they stimulate loading of sucrose into the phloem sap (Gajdanowicz *et al*., 2011). This mechanism exploits the differential operative status of the AKT2 K^+^ channel, which can partially replace the H^+^-ATPase when ATP is limited in availability (Dreyer *et al.*, 2017). However, little is currently known about the regulation of K^+^ uptake after the onset of anoxia.

In this work, we identified a CIPK protein, named CIPK25, which is involved in the regulation of K^+^ homeostasis under O_2_ shortage. CIPK25 is transcriptionally induced by low O_2_, preferentially in the root endodermis, and directly interacts with the inward rectifying K^+^ channel AKT1. Misregulation of CIPK25 under O_2_ shortage results in a lower K^+^ content in Arabidopsis seedlings, suggesting that this protein plays a role in maintaining ion homeostasis in these conditions.

## Materials and methods

### Plant material and growth conditions

The genotypes used were *Arabidopsis thaliana* ecotype Col-0 and Wassilewskija-2 (Ws-2), T-DNA insertion mutants *cipk25-2* (Col-0 SALK_070911c, previously isolated also by Meena *et al.*, 2019), *cipk25-3* (Col-0 SALK_059092), *cipk23-5* (Ragel *et al.*, 2015), *akt1-2* (Ws-2 NASC stock number: N3762; Nieves-Cordones *et al.*, 2019), and *akt1-1* (Hirsch *et al.*, 1998). The genetic status of the *cipk25-3* and *cipk25-2* lines was experimentally verified using primers listed in Supplementary Table S1 at *JXB* online. Homozygous plants for the CIPK25 locus were isolated in the *cipk25-2* and *cipk25-3* T-DNA insertion lines.

In order to visualize the CIPK25 promoter activity using the *GUS* and *GFP* reporter genes, a 1 kb genomic fragment corresponding to the 5′ region upstream of the gene was cloned and recombined into the *pKGWFS7* destination vector (Karimi *et al.*, 2002). The CIPK25 promoter was analyzed with AGRIS AtcisDB (https://agris-knowledgebase.org/AtcisDB) and PlantPAN 2.0 (http://plantpan2.itps.ncku.edu.tw). To overexpress the gene, the full-length coding DNA sequence of CIPK25 and the CIPK25*∆C* version, lacking the C-terminal domain, were amplified and recombined into the *pK7WG2* destination vector (Karimi *et al.* 2002). Transgenic plants were obtained using *Agrobacterium*-mediated transformation by the floral dip method (Clough and Bent, 1998). T_1_ seeds were screened on 0.9% agar plates containing the appropriate selective antibiotic. Resistant plants showing green cotyledons were screened until the T_3_ generation on selective medium.

To grow plants in pots, seeds were germinated in a moist soil mixture at 18–20 °C under a 12 h light photoperiod. The seeds were covered with plastic film for 1 week to maintain humidity. Seedlings were then transferred into new pots containing a growing mixture composed of soil, vermiculite, and fertilizer (ONE, Valagro). The plants were grown at 23 °C with a 12 h light photoperiod (120 μmol photons m^–2^ s^–1^) for 3 weeks.

To evaluate the plants’ submergence tolerance, plants were grown in pots for 3 weeks and then submerged in the dark for 72–96 h, starting the treatment at 20.00 h (the plants had been exposed to a 12 h light photoperiod with the light switched on at 08.00 h). The plants were then allowed to recover for 2 weeks.

**Fig. 1.**
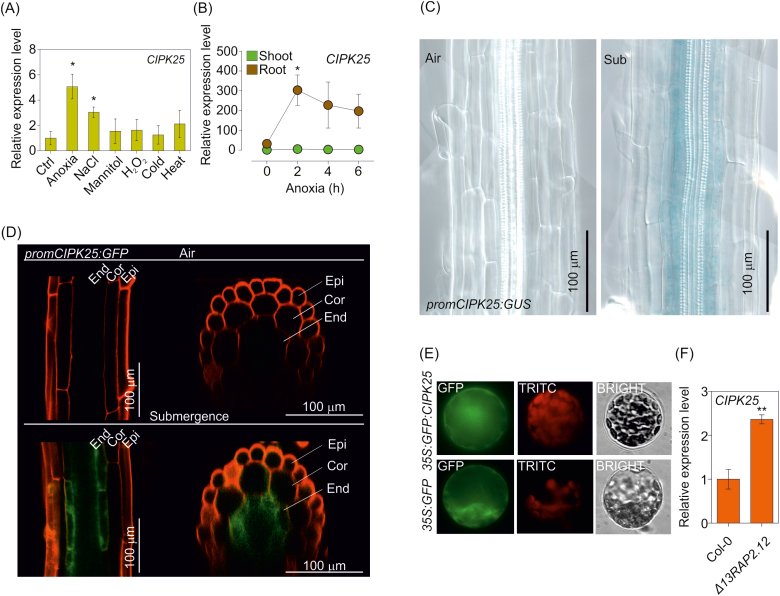
(A) Effect of abiotic stresses on the expression of *CIPK25*. Four-day-old seedlings were treated with 4 h of anoxia in darkness, 100 mM NaCl, 100 mM mannitol, 5 mM H_2_O_2_, 4 °C (cold), and 37 °C (heat). The value of the untreated control (Ctrl) was arbitrarily set to 1. Each value represents the mean ±SE (*n*=4). Statistical significance (Ctrl versus treatment) was determined using Student’s *t*-test: *P<0.05. (B) Time-course of *CIPK25* mRNA accumulation under anoxia in roots and shoots dissected from 3-week-old seedlings grown on vertical agar plates. Expression levels are shown as relative units, with the shoot value at time 0 arbitrarily set to 1. Each value represents the mean ±SE (*n*=3). Statistical significance (shoot versus root) was determined using Student’s *t*-test: *P<0.05. (C) Histochemical GUS staining of 10-day-old seedlings of the *promCIPK25:GUS* line under air and after 14 h of waterlogging (Sub). The experiment was performed in quadruplicate (*n*=15). (D) Confocal analysis of *promCIPK25:GFP* plants under air and after 14 h of waterlogging, showing longitudinal (left) and cross (right) sections along the z-axis. The experiment was performed in quadruplicate (*n*=10). (E) Confocal images of Arabidopsis protoplasts transiently transformed with *35S:GFP:CIPK25* and (as positive control) *35S:GFP* in air. (F) *CIPK25* mRNA accumulation in leaves of 3-week-old seedlings grown on vertical agar plates in Col-0 wild type and *Δ13rap2.12* (overexpressing a stable version of RAP2.12). The value of the wild type was arbitrarily set to 1. Each value represents the mean ±SE (*n*=3). Statistical significance was determined using Student’s *t*-test: **P<0.01.

For seedlings grown on six-well plates, a custom half-strength Murashige and Skoog (MS)-type medium, pH 5.7, was used, with the following recipe: 1.5 mM CaCl_2_, 0.75 mM MgSO_4_, 15 mM NH_4_NO_3_, 0.63 mM NH_4_H_2_PO_4_, 50 µM FeNaEDTA, 15 µM ZnSO_4_, 0.5 µM Na_2_MoO_4_, 50 µM MnSO_4_, 5 µM KI, 50 µM H_3_BO_3_, 0.05 µM CoCl_2_, and 0.5 µM CuSO_4_, with KCl added at 10, 2.5, or 0.1 mM. In Fig. 1A, MS (Duchefa Biochemie, product number M0221) was used. Seedlings were stratified in the dark at 4 °C for 2 days and then grown at 23 °C with a 12 h light photoperiod for 3 days before the dark-anoxia treatment. This treatment was applied in an enclosed anaerobic workstation (1 Person Hypoxic Glove Box, Coy Laboratory Products).

For gene expression analysis, seedlings were harvested directly after the treatment. For quantification of chlorophyll content and K^+^ content, seedlings were allowed to recover in the growth chamber for 1 additional week after the treatment before analysis.

For measurement of stalk height, plants were grown in pots for 3 weeks, then waterlogged for an additional 3 weeks before being measured with a ruler. To overcome differences in the vegetative to flowering transition between Col-0 and Ws-2 wild-type plants, both neutral (12 h light/12 h dark) and long-day (16 h light/8 h dark) conditions were evaluated, since in long-day conditions Ws-2 showed the phenotype after 5 days of waterlogging.

### Isolation and transfection of Arabidopsis protoplasts

Arabidopsis Col-0 mesophyll protoplasts were obtained from leaves of 3-week-old plants grown in a plastic pot filled with soil and peat (3:1) at 25/20 °C day/night under a 12 h light photoperiod, with photosynthetically active radiation of 100 µmol m^–2^s^–1^ provided by fluorescence lamps. Protoplasts were isolated as previously described (Yoo *et al.*, 2007) and transformed with polyethylene glycol, using 5 µg of each plasmid. Protoplasts were incubated for 16 h at 25 °C in the dark and then immediately visualized.

### RNA extraction and real-time PCR analysis

Total RNA was extracted as previously described (Kosmacz *et al.*, 2015). Total RNA was reverse transcribed using the Maxima First Strand cDNA synthesis kit (Thermo Scientific). Real-time PCR reactions were carried out using SYBR® Green PCR Master Mix (Bio-Rad Laboratories, USA), using specifically designed primers (see Supplementary Table S1). The ∆∆Ct method was applied for relative quantification (Livak and Schmittgen, 2001).

### Localization of GUS/GFP in plants and protoplasts

Protoplasts transfected with a *35S:GFP:CIPK25* construct were observed with a Nikon Eclipse Ti-5 ViCo epi-fluorescence microscope (Nikon, Japan) using GFP and TRITC filters. The *pAVA* vector (von Arnim *et al.*, 1998) was used as a 35S:GFP control. For bimolecular fluorescence complementation (BiFC) experiments, protoplasts were visualized with a ZEISS LSM880 Airyscan confocal microscope. Yellow fluorescent protein (YFP) fluorescence was excited at 488 nm and collected at between 520 and 560 nm. Chlorophyll autofluorescence was excited at 633 nm and collected at between 650 and 750 nm. Images were analyzed with ZEN 2010 software (Zeiss).

*PromCIPK25*-GUS/GFP seeds were plated on half-strength MS medium and seedlings were grown vertically in long-day conditions (16 h light/8 h dark) as previously described (Di Mambro and Sabatini, 2018). At 7 days after germination, seedlings were submerged by direct injection of distilled water into the plate. Plants were submerged vertically up to the level of the hypocotyl/root junction. After 14 h of being submerged, water was drained from the plate and the plants were analyzed by confocal microscopy. For visualization of cells, seedlings were directly mounted on a slide in 10 µl of propidium iodide (PI) solution (10 μg ml^–1^ dissolved in water) to stain the cell wall; PI and GFP fluorescence were acquired using a 488 nm laser and a highly sensitive gallium arsenide phosphide (GaAsP) spectral photodetector [EC Plan-Neofluar ×40/1.30 Oil differential interference contrast (DIC) objective, pinhole 90 µm]. DIC microscopic analysis followed GUS histochemical staining performed as described in Vitha *et al.* (1995).

### Cloning and protein–protein interaction assays

Coding and regulatory *CIPK25* sequences were amplified from Arabidopsis Col-0 genomic DNA template using Phusion High Fidelity DNA polymerase (New England Biolabs, UK) following the manufacturer’s instructions and using the primers listed in Supplementary Table S1. The PCR products were purified and cloned into the Gateway pENTR/D-TOPO vector (Life Technologies, USA). The resulting entry clones were recombined into destination vectors using the LR Reaction Mix II (Life Technologies, USA). Each cloning product was verified by restriction-site mapping and sequencing. For the BiFC experiments, the *CIPK25∆C* sequence, consisting of the kinase domain, and the C-terminal cytosolic domain of *AKT1* were cloned into the pDH51-GW-YFPC or pDH51-GW-YFPN vector (Zhong *et al.*, 2008), respectively, and co-transfected into Arabidopsis mesophyll protoplasts. The tag orientation was defined in line with previous results (Xu *et al.*, 2006). Corresponding empty vectors were used as negative controls. pAVA (35S:GFP) (von Arnim *et al.*, 1998) was used as a positive control of transformation.

For yeast two-hybrid (Y2H) analysis, the *CIPK25∆C* sequence and the C-terminal cytosolic domain of *AKT1* were cloned into the pDEST32 or pDEST22 vector (ThermoFisher), respectively. Co-transformation was performed in the MaV203 yeast strain following the Li/Ac protocol (ThermoFisher Proquest). Positive colonies for transformation were screened in SD-LT medium and interaction was screened in SD-LTUH+15 mM 3AT medium (Sigma-Aldrich). Empty vectors were used as negative controls. X-gal staining was performed on the same plates with filter paper soaked in Z-buffer (ThermoFisher) and left at 37 °C for 2–3 h. Homodimerization of HRU1 (Gonzali *et al.*, 2015) was used as a positive control.

### Total chlorophyll content analysis

Chlorophyll extraction was performed in the dark, using ethanol (96% v/v) as a solvent. The samples were incubated at 4 °C overnight. After centrifugation (11 200 *g* for 5 min at 4 °C), total chlorophyll content was measured spectrophotometrically using the formula of Lichtenthaler and Buschmann (2001).

### Quantification of potassium content

To quantify the K^+^ content, seedlings grown as described above were collected, dried in an oven at 60 °C for 10 days, and their dry weight was recorded. They were then mineralized in 65% HNO_3_ at 200 °C in an Anton Paar Multiwave 7000 microwave and finally analyzed in a Bruker AURORA ICP-MS mass spectrometer.

## Results

### CIPK25 is induced in Arabidopsis roots under oxygen shortage

Among the 26 CIPKs encoded by the Arabidopsis genome, *CIPK25* (*At5g25110*) is transcriptionally induced under low-O_2_ conditions (Supplementary Fig. S1), pointing to a putative role for this kinase under this stress condition. Gene expression analysis in Arabidopsis seedlings exposed to different abiotic stresses identified *CIPK25* as a salt- and anoxia-induced gene (Fig. 1A), preferentially in roots (Fig. 1B). *CIPK25* expression was also detected in 10-day-old seedlings using *promCIPK25:GUS* (Fig. 1C) and *promCIPK25:GFP* (Fig. 1D) plants, revealing a preferential induction under waterlogging, localized in the root endodermis. Observation of mesophyll protoplasts transiently transformed with the *35S:GFP:CIPK25* construct revealed that CIPK25 protein is preferentially localized in the cytosol (Fig. 1E). Bioinformatic inspection of the *CIPK25* promoter identified the presence of several *MYB*, *ABRE*, and *ARF* binding motifs (Supplementary Fig. S2A). However, the hypoxia-responsive promoter element (*HRPE*), which is responsible for the regulation of core anaerobic genes (Gasch *et al.* 2016), was absent. Interestingly, a GCC-box (position –138), a known target of AP2/ERF transcription factors (Yang *et al.*, 2009; Lee *et al.*, 2015), was also found (Supplementary Fig. S2B). In line with this observation, *CIPK25* is expressed at a higher level in plants overexpressing a chimeric form of RAP2.12 lacking the first 13 N-terminal amino acids containing the destabilizing Cys_2_ (*35S:Δ13RAP2.12*; Giuntoli *et al.*, 2017) (Fig. 1F).

### CIPK25 is involved in tolerance to hypoxia

**Fig. 2.**
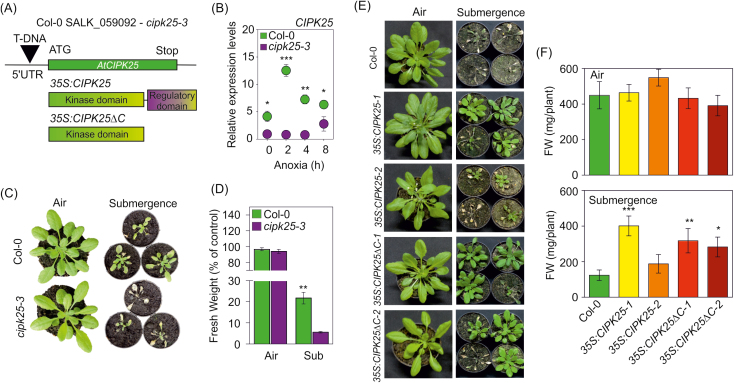
(A) T-DNA insertion in the Arabidopsis mutant *cipk25-3* (background Col-0) and detail of the *CIPK25* and *CIPK25ΔC* constructs in transgenic overexpressing lines. (B) Gene expression analysis of the relative expression level of *CIPK25* in Col-0 and *cipk25-3* mutant roots of 21-day-old plants grown on vertical plates. The value for *cipk25-3* at time 0 was arbitrarily set to 1. Each value represents the mean ±SE (*n*=4). Statistical significance (*cipk25-3* versus Col-0) was determined using Student’s *t*-test: *P<0.05, **P<0.01, ***P<0.001. (C, D) Effect of 72 h of submergence in the dark on the survival of the *cipk25-3* Arabidopsis mutant; data presented are the mean ±SE of shoot fresh weight measurements, expressed as a percentage of shoot fresh weight for the control in air (*n*=3). Statistical significance (*cipk25-3* versus Col-0) was determined using Student’s *t*-test: **P<0.01. (E, F) Effect of 96 h of submergence in the dark on the survival of the *CIPK25-1*, *CIPK25-2, CIPK25ΔC-1*, and *CIPK25ΔC-1* overexpressing lines; Data presented are the mean ±SE (*n*=10) shoot fresh weight measurements. Statistical significance (all genotypes versus Col-0) was determined using Student’s *t*-test: *P<0.05, **P<0.01, ***P<0.001. Controls were grown under a photoperiodic regime.

In order to assess the involvement of CIPK25 under low-O_2_ conditions, we examined the response of a T-DNA insertional mutant, *cipk25-3*, to being submerged. This line has a T-DNA insertion in the 5′ untranslated region of the gene (Fig. 2A), which abolished gene induction in roots under anoxia (Fig. 2B). *cipk25-3* mutant plants exposed to 72 h of submergence in the dark showed poorer survival after recovery compared with wild-type plants (Fig. 2C, D). Similar results were obtained using the *cipk25-2* mutant line (Supplementary Fig. S3; Meena *et al.*, 2019). The effect of submergence was also assessed in plants overexpressing *CIPK25*. To do this, we generated four transgenic lines, in which the full gene (*CIPK25-1*, *CIPK25-2*) or a truncated version containing only the kinase domain and lacking the autoinhibitory domain (*CIPK25ΔC-1*, *CIPK25ΔC-2*) (Chaves-Sanjuan *et al.*, 2014) were under the control of the *CaMV 35S* promoter (Fig. 2A, Supplementary Fig. S4). Previous results showed that, in the absence of the CBL partner, the activity of the CIPK is higher when the C-terminal domain is removed (Lee *et al.*, 2007). The overexpressing lines (both full and truncated versions of CIPK25) showed a similar phenotype under aerobic conditions, comparable with previous observations (Meena *et al.*, 2019). Overexpression of the two *CIPK25ΔC* lines and *CIPK25-1* conferred enhanced tolerance to submergence compared with the wild type after 96 h of submergence in darkness (Fig. 2E, F). When grown on vertical plates, the *35S:CIPK25-1* and *35S:CIPK25ΔC* transgenic lines showed longer roots, while the roots of *cipk25-3* were significantly shorter than those of wild-type plants (Supplementary Fig. S5), as previously observed (Meena *et al.*, 2019).

### CIPK25 interacts with the AKT1 potassium channel

**Fig. 3.**
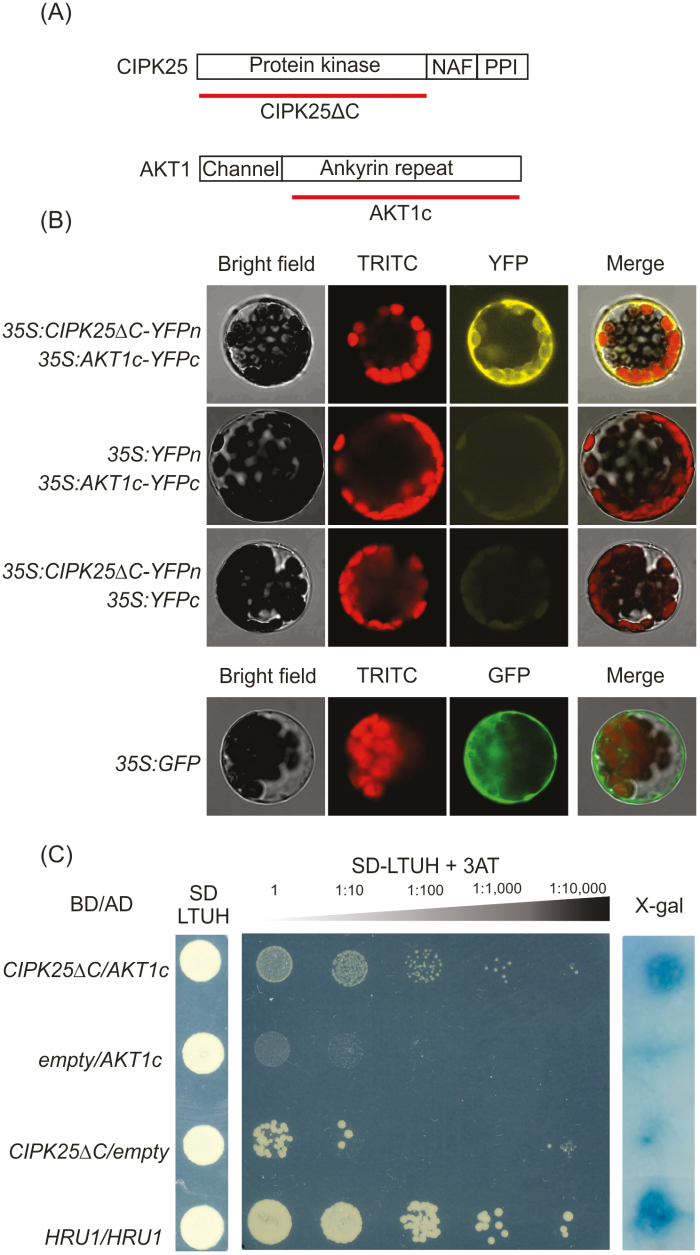
(A) Constructs used for bimolecular fluorescence complementation (BiFC) and yeast two-hybrid (Y2H) screening for protein–protein interaction. (B) BiFC assay demonstrating the interaction between CIPK25ΔC and the AKT1 C-terminus (AKT1c). Alternate empty vectors were used as negative controls. The pAVA vector was used as a 35S:GFP positive control of transformation. The experiment was run in triplicate. (C) Y2H assay demonstrating the interaction between CIPK25ΔC and AKT1c. Yeast strains were initially plated to an OD_600_ of 0.8, and at serial 10-fold dilutions on selective medium (SD-LTUH) supplemented with 3-AT (15 mM). Alternate empty vectors were used as negative controls. X-gal staining of colonies was used to further confirm the interaction. The homodimerization of HRU1 (Gonzali *et al.*, 2015) was used as a positive control. Three independent colonies were screened for interaction.

In order to identify putative interacting partners of CIPK25, we focused on the localization of the protein in the root endodermis (Fig. 1D) and the established role of the CIPK family as a regulator of channels and transporters. We compared the expression patterns of *CIPK25* with those of the K^+^ channels *AKT1* and *AKT2* by using the Genevestigator (https://genevestigator.com/gv/) and Arabidopsis Translatome eFP (http://efp.ucr.edu/) browsers (Supplementary Fig. S6). Under O_2_ shortage, at the organ and tissue level, expression of *CIPK25* and *AKT1* converges in specific cell types in the root (Supplementary Fig. S6A), above all the cortex and the endodermis (Supplementary Fig. S6B). In order to test the interaction between CIPK25 and AKT1, the ankyrin repeat domain of AKT1 and the kinase domain of CIPK25 were used (Fig. 3A). Using BiFC, we found that CIPK25ΔC physically interacts with the C-terminal cytosolic domain of the inward K^+^ channel AKT1 (AKT1c; Fig. 3B); we did not find any interaction with AKT2 (Supplementary Fig. S7). The CIPK25ΔC–AKT1c interaction was also confirmed by Y2H analysis using auxotrophic selection and X-gal staining (Fig. 3C). Previous results showed that the kinase domain of CIPK23 is responsible for the interaction with the ankyrin repeat domain of AKT1 (Lee *et al.*, 2007). Moreover, only the kinase domain of CIPK23 is able to enhance the channel activity of AKT1 in absence of the CBL partner (Lee *et al.*, 2007). However, when testing the tolerance of the *cipk23-5* mutant to submergence stress we did not observe any phenotype (Supplementary Fig. S8).

### CIPK25 regulates potassium homeostasis under anoxia

**Fig. 4.**
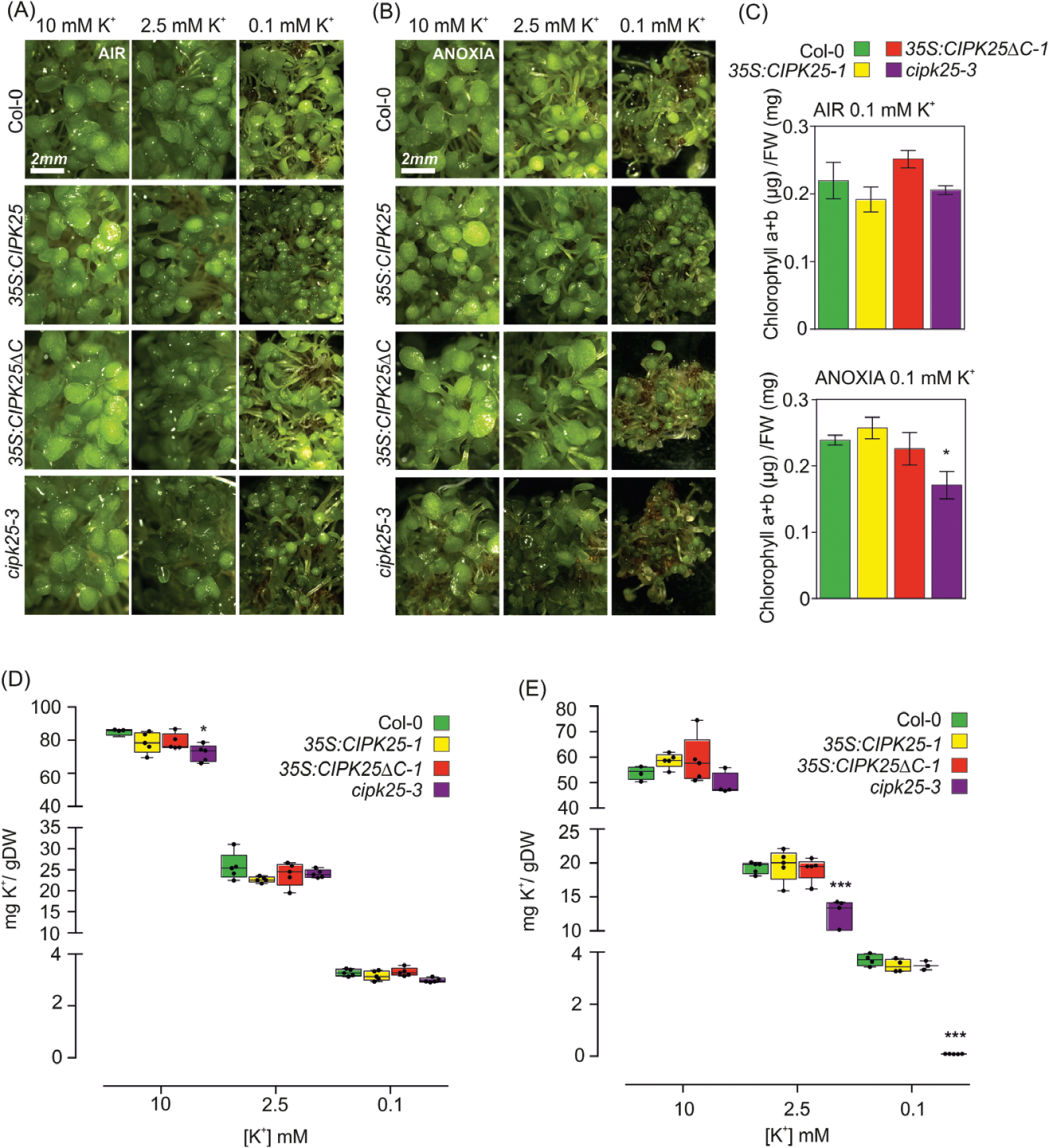
Phenotype of Col-0, *cipk25-3* mutant, and *CIPK25* and *CIPK25ΔC* overexpressing seedlings grown in different K^+^ concentrations in air (A) and with exposure to 8 h of anoxia followed by 1 week of recovery (B). (C) Total chlorophyll content (mean ±SD, *n*=3) in plants grown with 0.1 mM K^+^ under air or anoxia followed by 1 week of recovery. (D, E) K^+^ concentration (mean ±SE, *n*=5) of Col-0, *cipk25-3* mutant, and *CIPK25 and* CIPK25ΔC overexpressing seedlings grown under different K^+^ concentrations in air (D) and under 8 h of anoxia followed by 1 week of recovery (E), with relative box plots. Statistical significance (genotypes versus Col-0) was determined using Student’s *t*-test: *P<0.05, ***P<0.001.

In order to understand the interplay between low O_2_ and K^+^, Arabidopsis Col-0, the *cipk25-3* mutant, and *CIPK25-1* and *CIPK25ΔC-1* overexpressing seedlings grown in the presence of different K^+^ concentrations were exposed to 8 h of anoxia or maintained in aerobic conditions. When the K^+^ content in the medium was at a concentration of 0.1 mM, seedling growth was reduced irrespective of the anoxic treatment (Fig. 4A). When plants were grown with only 0.1 mM K^+^, the ability of the *cipk25-3* mutant plants to recover after anoxia was more severely affected compared with the other lines (Fig. 4B). The total chlorophyll content of seedlings exposed to 0.1 mM K^+^ and anoxia was significantly higher in control and *CIPK25-1* overexpressing seedlings in comparison to *cipk25-3* mutant plants (Fig. 4C).

We then quantified the K^+^ content in Arabidopsis Col-0, *cipk25-3* mutant, and *CIPK25-1* and *CIPK25ΔC-1* overexpressing seedlings. While only a small reduction in K^+^ content was observed in the *cipk25-3* mutant grown in 10 mM K^+^ medium under aerobic conditions relative to the other genotypes analyzed (Fig. 4D), *cipk25-3* seedlings grown in the 2.5 and 0.1 mM K^+^ media and exposed to anoxia followed by recovery showed a significant reduction in K^+^ content compared with all the other genotypes (Fig. 4E). We also quantified the K^+^ content of *akt1-1* and *akt1-2* mutants in comparison to their wild-type background (Ws-2 and Col-0, respectively), but no differences were found in seedlings grown in either aerobic or anoxic conditions (Supplementary Fig. S9).

**Fig. 5.**
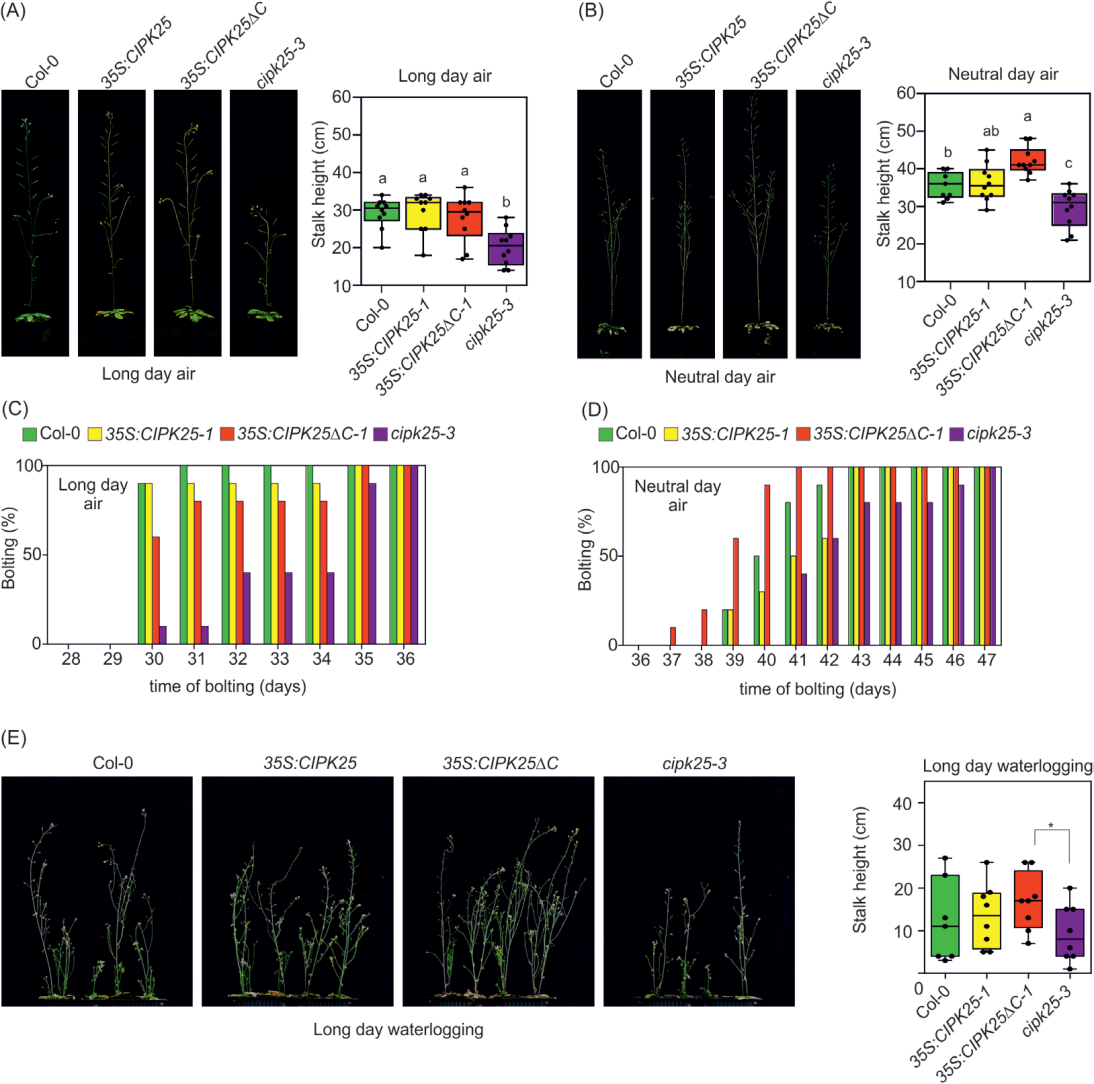
Phenotype of Col-0, *cipk25-3* mutant, and *CIPK25* and *CIPK25ΔC* overexpressing plants grown under long (A, C) and neutral (B, D) photoperiod conditions in air, with plots of percentage bolting by day and final stalk height (mean ±SE, *n*=10). Statistical significance was determined using two-way ANOVA test followed by a Tukey post-hoc test; significant differences (*P*<0.001) are indicated in the box plots with different letters. (E) Phenotype of Col-0, *cipk25-3* mutant, and *CIPK25* and *CIPK25ΔC* overexpressing plants grown under long-day conditions under submergence, with final stalk height depicted in box plots (mean ±SE, *n*=8). Statistical significance was determined using Student’s *t*-test: *P<0.05.

**Fig. 6.**
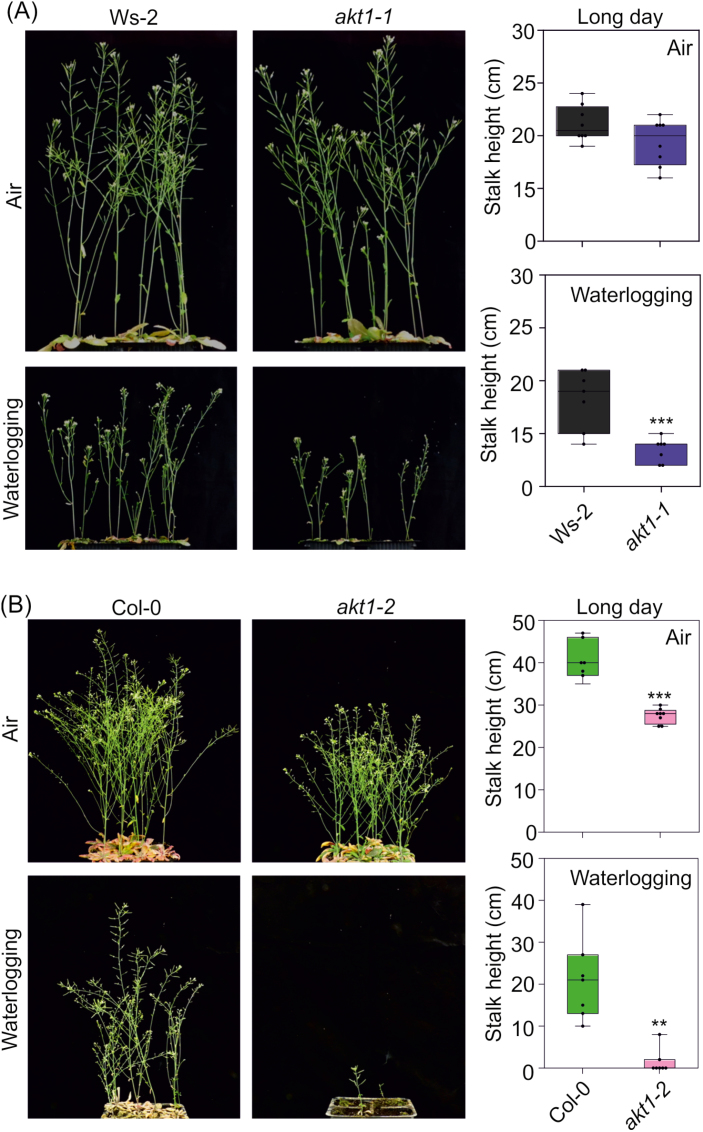
(A) Phenotype of Ws-2 and *akt1-1* plants grown in air or waterlogging under long-day conditions, with final stalk height depicted in box plots. (B) Phenotype of Col-0 and *akt1-2* plants grown in air and with waterlogging under long-day conditions, with final stalk height depicted in box plots. Statistical significance (mean ±SD, *n*=8) was determined using Student’s *t*-test: **P<0.01, ***P<0.001.

Mutants with impaired K^+^ homeostasis showed reduced stalk height and a delay in bolting under energy-limiting conditions (Gajdanowicz *et al.*, 2011; Held *et al.*, 2011; Sklodowski *et al.*, 2017). This phenotype was previously observed in *akt2-1* mutant plants grown under neutral and short-day photoperiods and in the presence of low O_2_ under a long-day photoperiod (Gajdanowicz *et al.*, 2011). We observed a reduced stalk height in the *cipk25*-3 mutant under both long (16 h/8 h light/dark) and neutral (12 h/12 h light/dark) photoperiod regimes (Fig. 5A, B). Moreover, in both growth conditions, *cipk25-3* showed a delay in bolting (Fig. 5C, D). This phenotype was also observed when plants were exposed to waterlogging under long-day conditions (Fig. 5E). Under neutral-day conditions and waterlogging, all analyzed genotypes in the Col-0 background failed to induce stalk elongation and senesced (data not shown). By contrast, the *akt1-1* and Ws-2 lines showed a similar stalk height when grown in air under the long-day regime; under waterlogging, plants showed a significant difference in stalk height (Fig. 6A). Another mutant, *akt1-*2 (Col-0 background), showed a significant reduction in stalk height compared with Col-0 under both air and waterlogging, with waterlogged *akt1-2* plants more strongly affected (Fig. 6B).

## Discussion

In roots, the reduction of energy availability resulting from the inhibition of respiration under anaerobic conditions has a direct impact on the activity of membrane transporters, which in turn interferes with the nutrient-acquisition capacity of the plant (Shabala *et al.*, 2014). Moreover, under O_2_ deprivation plasma membrane depolarization occurs (Buwalda *et al.*, 1988; Teakle *et al.*, 2013; Zeng *et al.*, 2014), leading to an imbalance in ion homeostasis.

Under O_2_ shortage, K^+^ uptake is strongly reduced (for a review see Elzenga and van Veen, 2010). The plant’s capacity to maintain K^+^ cytosolic homeostasis together with the functionality of the K^+^ channels has been proposed as an essential adaptation for plant survival under hypoxia (Mancuso and Marras, 2006; Mugnai *et al.*, 2011). In barley, the extent of K^+^ loss has been shown to be proportional to O_2_ availability, with anoxic plants showing a more profound K^+^ deprivation in comparison to hypoxic plants (Zeng *et al.*, 2014). Arabidopsis *gork1-1* mutants, which lack a functional K^+^ efflux channel, have been shown to be highly tolerant to hypoxia (Wang *et al.*, 2017*a*), suggesting that the ability of plants to retain K^+^ is involved in stress tolerance.

The capacity to finely regulate K^+^ homeostasis under different stress conditions, for example, salt stress (reviewed by Shabala and Pottosin, 2014), oxidative (Demidchik *et al.*, 2014), and heavy metal contamination (Murphy and Taiz, 1997), is a common feature of stress-tolerant plants. K^+^ is also a possible second messenger in plant stress adaptation, likely activating the shift toward a plant defense state (Shabala, 2017). Under O_2_ shortage, K^+^ may play a role in restoring membrane potential after low O_2_-dependent depolarization. Moreover, K^+^ loss also represents a metabolic controller (Demidchik *et al.*, 2014), since K^+^-dependent enzymes can be inactivated and the ATP pool preserved (Shabala, 2017).

Depending on the availability of K^+^ in the soil, different K^+^ uptake systems, ranging from low to high affinity, are active (Sharma and Sharma, 2013). The AKT1 channel is involved in low-affinity (>5 mM) to high-affinity (up to 0.1 mM) K^+^ uptake and is the target of an extensive regulatory network that includes CBL Ca^2+^ sensors and CIPK proteins. In normoxia, AKT1 is regulated by members of the CIPK family, which mediate the transition of the channel from low to high affinity upon phosphorylation (Xu *et al.*, 2006). In this context, CIPK23, in association with CBL1 and CBL9, enhances K^+^ uptake in Arabidopsis under low-K^+^ conditions (Li *et al.*, 2006; Xu *et al.*, 2006).

The action of AKT1 is almost confined to the root, in line with its function as a K^+^ uptake channel. Nonetheless, *AKT1* expression increases under hypoxic conditions, mainly in the elongation zone of the root (Supplementary Fig. S6), suggesting that K^+^ uptake mechanisms may play a role under the stress (Shabala, 2017). It is of note that at the tissue level, the expression of *CIPK25* and *AKT1* converges in the root endodermis (Supplementary Fig. S6), supporting a putative interplay between these two proteins *in vivo* in this tissue.

In this context, endodermal cells have the ability to develop a localized deposit of lignin polymers in the radial and transverse section (the Casparian strip), highlighting the importance of this tissue as a checkpoint for nutrient homeostasis (for a review see Barberon and Geldner, 2014). This secondary cell-wall modification interrupts the apoplastic diffusion route of solutes and water to the stele, such that the transport of nutrients is allowed only through channels or plasmodesmata to the inner vasculature (for a review see Barberon, 2017). Pfister *et al.* (2014) characterized a receptor-kinase mutant, *schengen3*, involved in Casparian strip positioning and showing defects in K^+^ homeostasis. In addition, the use of bioimaging at the cellular level in Arabidopsis roots highlighted a high concentration of K^+^ in the central vasculature, making the endodermis the ultimate checkpoint for the efficient loading of K^+^ into the inner tissues and its redistribution in other organs (Persson *et al.*, 2016).

A dramatically lower vascular K^+^ concentration was found in stagnant barley roots in comparison to aerated roots (Zeng *et al.*, 2014), highlighting the importance of efficient regulation of K^+^ uptake under hypoxia to allow K^+^ loading into the xylem sap. More recently, it has also been shown that AKT1 may play a role in the retrieval of K^+^ from xylem vessels (Nieves-Cordones *et al.*, 2019). In this context, the up-regulation of *CIPK25* in the endodermis under low O_2_ (Fig. 1C, D) may underlie an adaptive mechanism to preserve the efficiency of K^+^ accumulation in this tissue, whereby K^+^ could be translocated to the vasculature and consequently distributed throughout the whole plant. Given that the inner tissues of the root may have an endogenous reduced diffusion of O_2_ (Shukla *et al.*, 2019), this hypothesis is in agreement with the final lower stalk height of the *cipk25-3* mutant grown in air (Fig. 5A, B) and with the lower K^+^ content found in *cipk25-3* grown in air in the presence of 10 mM K^+^ in the medium (Fig. 4D).

Among the CIPK family, we found that *CIPK25* is positively regulated at the transcriptional level by O_2_ deficiency in roots (Supplementary Fig. S1, Fig. 1A–D). *CIPK25* is also up-regulated in the *ate1-2* mutant in comparison to wild-type Col-0, in *HRE1* and *HRE2* overexpressing plants under low O_2_ (Supplementary Fig. S1), and in *35S:Δ13RAP2.12* transgenic plants (Fig. 1F). It might thus be possible that either *CIPK25* is a target of RAP2.12 or that HRE1 and HRE2 are responsible for *CIPK25* expression under low O_2_. In fact, a known target of AP2/ERF transcription factors, a GCC-box, is present in the *CIPK25* promoter (Supplementary Fig. S2). Alternatively, *CIPK25* expression might be indirectly affected by the O_2_ sensing machinery, downstream of RAP2.12.

Overall, the activation of a CBL–CIPK sensor relay complex, post-translationally regulated by Ca^2+^, suggests a mechanism in which low O_2_ and the presence of Ca^2+^ spiking converge in protecting the plant from K^+^ leakage. We noticed that CIPK23, which is known to regulate AKT1 under K^+^ starvation (Li *et al.*, 2006; Xu *et al.*, 2006), seems not to be involved in adaptation to submergence, since the survival of the *cipk23-5* mutant was similar to that of the Col-0 control (Supplementary Fig. S8). This suggests that the possible impairment in K^+^ homeostasis that occurs under anoxia may fail to activate the mechanism that in air enhances the capability for K^+^ uptake—that is, disruption in K^+^ homeostasis-activated Ca^2+^ spiking, activation of CBL1/9 by Ca^2+^, interaction of CBL1/9 with CIPK23, phosphorylation of AKT1 by CIPK23, and transition of AKT1 from low to high affinity (Li *et al.*, 2006; Xu *et al.*, 2006; Cheong *et al.*, 2007; Lee *et al.*, 2007). The result of low-O_2_-dependent induction of *CIPK25* may compensate for this impairment by activating the K^+^ channel AKT1. Y2H and BiFC results (Fig. 3) identified the presence of an interaction between CIPK25 and AKT1, supporting this hypothesis. Recently, CIPK25 has been found to interact with CBL4 (Meena *et al.*, 2019), which is also strongly expressed in Arabidopsis roots under hypoxia (eFP Translatome browser; data not shown), suggesting a post-translational activation mechanism mediated by Ca^2+^ under low O_2_. The presence of an early Ca^2+^ spike under low O_2_ has been recently confirmed using a FRET-based biosensor (NES-YC3.6) (Wagner *et al.*, 2019).

The activation of CIPK25 may be a prerequisite for AKT1 functioning under combined O_2_ shortage and low K^+^, as suggested by the strong reduction in K^+^ content in the *cipk25-3* mutant under anoxia and low K^+^ content in the medium (Fig. 4E). Indeed, the reduction in cellular K^+^ concentration in the *cipk25-3* mutant was observed exclusively in media with low K^+^ concentrations (2.5 and 0.1 mM K^+^) where almost only high-affinity K^+^ channels, such as AKT1, play a role.

AKT1 is probably not the only K^+^ uptake mechanism functioning under O_2_ shortage, since the *akt1-1* and *akt1-2* mutants did not show a strong reduction in K^+^ content relative to their respective wild type (Supplementary Fig. S9). The mechanism of K^+^ uptake under low O_2_ by CIPK25 likely includes some other transporters, which have not yet been identified.

Interestingly, *cipk25* mutant lines have a reduced root length (Supplementary Fig. S5; Meena *et al.*, 2019) and altered auxin transport, possibly due to misregulated PIN protein expression (Meena *et al.*, 2019). Philippar *et al.* (2006) reported that the application of exogenous auxin transcriptionally regulates the *Zea mays* inward K^+^ channel ZMK1. Moreover, Arabidopsis AKT1 is involved in the sensing of external K^+^ concentration, with a subsequent regulation of PIN protein abundance and auxin redistribution in roots (Li *et al.*, 2017). In fact, mutants for K^+^ efflux channels, which likely have a higher concentration of K^+^ in the cytosol, show increased cell expansion, likely connected to auxin (Osakabe *et al.*, 2013). It thus seems that in aerobic conditions an interplay between CIPK25, AKT1, and auxin might occur in Arabidopsis roots in order to regulate growth, a mechanism that deserves further investigation.

Our results show that CIPK25 plays a key role in maintaining K^+^ homeostasis under low-O_2_ conditions. This mechanism is transcriptionally regulated by low O_2_ and likely by Ca^2+^-dependent signaling at the post-translational level. In addition to AKT1, other targets of CIPK25 could be involved in leading to adaptive responses that modify K^+^ fluxes not only under environmental low O_2_ but also under endogenous tissue-specific hypoxia.

## Supplementary data

Supplementary data are available at *JXB* online.

Table S1. List of primers.

Fig. S1. Expression of *CIPK25* under low O_2_ conditions and different genetic backgrounds related to hypoxia.

Fig. S2. *CIPK25* promoter analysis through AGRIScisDB platform and PlantPAN2.0.

Fig. S3. Phenotype of *cipk25-2* mutant under submergence stress.

Fig. S4. *CIPK25* expression level in overexpressing plants. 

Fig. S5. Root length of seedlings grown on plates.

Fig. S6. Comparison between *CIPK25*, *AKT1* and *AKT2* expression pattern under O_2_ shortage in various Arabidopsis tissues using Genevestigator software and eFp Translatome Browser.

Fig. S7. BiFC assay showing no interaction between CIPK25 and AKT2.

Fig. S8. Effect of submergence on the survival of *cipk23-5* Arabidopsis mutants.

Fig. S9. Potassium cellular concentration of Ws-2 and *akt1-1* and Col-0 and *akt1-2* seedlings grown under different external K^+^ concentrations in air and anoxia.

eraa004_suppl_Supplementary_Materials
