# Supplementary material for: The calcineurin β-like interacting protein kinase CIPK25 regulates potassium homeostasis under low oxygen in Arabidopsis
Source: J Exp Bot. 2020 Feb 13;71(9):2678–89. doi: 10.1093/jxb/eraa004 (PMC7210770; doi:10.1093/jxb/eraa004)
Supplement: eraa004_suppl_Supplementary_Materials [file eraa004_suppl_supplementary_materials.pdf]

SUPPLEMENTARY DATA

Table S1 – List of primers used in this study.

Gene ID and name	Forward primer (5'-3')	Reverse primer (5'-3')
At5g25110 (CIPK25) RT-qPCR	GTCGTGGCACGGTGATAAT	ACATTCTCTTAGCAGTCACTACCAG
N-termCIPK25 RT-qPCR	GGAGTTTGAGTATCCGCCGT	TGTTCGCATAATCGCCGGTA
At4g05320 (UBQ10 RT-qPCR	GGCCTTGTATAATCCCTGATGAATAAG	AAAGAGATAACAGGAACGGAAACATAGT
cdsCIPK25	CACCATGGGATCCAAACTTAAAC	ACATTCTCTTAGCAGTCACTACCAG
CIPK25ΔC	CACCATGGGATCCAAACTTAAAC	TCACTTCGGCGAGACCGGTGT
promCIPK25	CACCTCAACATTTAAGGATTTCTA	GTGTATATACAGAAGTAGAATGAG
C-termAKT1	CACCATGACAAATTTGGTAGTCCATGGA	AGAATCAGTTGCAAAGATGAGATGAT
attB1	GGGACAAGTTTGTACAAAAAAGCAGCTG	
attB2		GGGACCACTTTGTACAAGAAAGCTGGGT
cipk25-3 (mutant screening)	GCCTCATACATCCCCTCTCA	GTTTAAGTTTGGATCCCATGTGT
cipk25-2 (mutant screening)	TGGTCTCTCTGCTTTACCGG	CACCGCGATCTGTCCTTTC
LBb1	GCGTGGACCGCTTGCTGCTG	

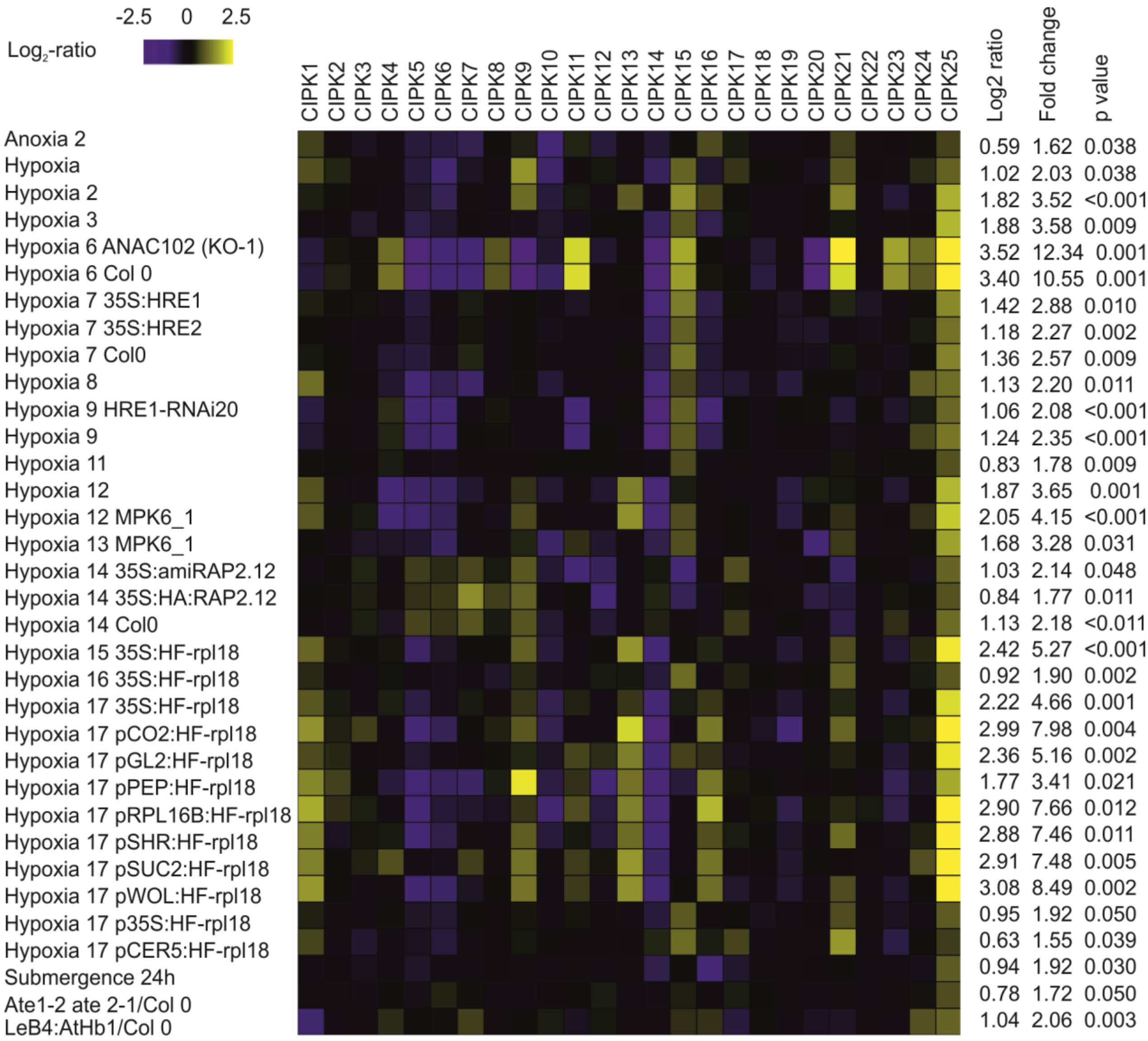

Figure S1. Significant expression of *Calcineurin-B -Interacting Protein Kinase 25* (*CIPK25*, Fold change>1.5, p-value<0.05) under low O_2 conditions and different genetic backgrounds related to hypoxia. Available datasets of microarray analyses performed under various conditions of low O_2 stress were selected and queried using Genevestigator (https://genevestigator.com/gv/). *CIPK26* was not represented on the chosen platform. The genotypes significantly affected correspond to: *ate1-2ate2-1*, a mutant for the Arg-tRNA protein transferase (ATE) impaired in the N-end pathway for target proteasomal degradation under O_2 shortage (Gibbs et al., 2011; Licausi et al., 2011); and *LeB4:AtHb1*, carrying the coding region of *AtHb1* (a non-symbiotic hemoglobin of class 1 with superior affinity for O_2) under the control of the seed-specific *LeB4* promoter (Thiel et al., 2011).

(A)

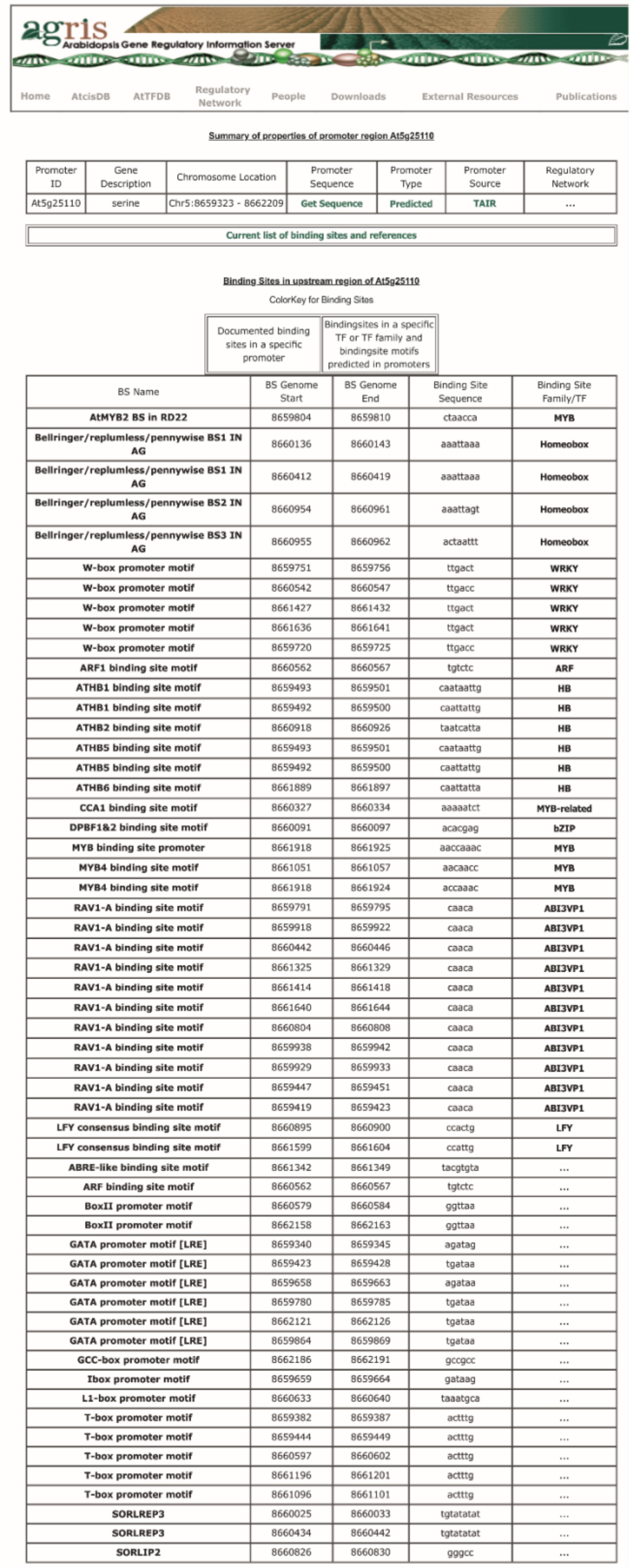

Summary of properties of promoter region At5g25110

Promoter ID	Gene Description	Chromosome Location	Promoter Sequence	Promoter Type	Promoter Source	Regulatory Network
At5g25110	serine	Chr5:8659323 - 8662209	Get Sequence	Predicted	TAIR	...

Current list of binding sites and references

Binding Sites in upstream region of At5g25110

ColorKey for Binding Sites

Documented binding sites in a specific promoter	Bindingsites in a specific TF or TF family and bindingsite motifs predicted in promoters

BS Name	BS Genome Start	BS Genome End	Binding Site Sequence	Binding Site Family/TF
AtMYB2 BS in RD22	8659804	8659810	ctaacca	MYB
Bellringer/replumless/pennywise BS1 IN AG	8660136	8660143	aaattaaa	Homeobox
Bellringer/replumless/pennywise BS1 IN AG	8660412	8660419	aaattaaa	Homeobox
Bellringer/replumless/pennywise BS2 IN AG	8660954	8660961	aaattagt	Homeobox
Bellringer/replumless/pennywise BS3 IN AG	8660955	8660962	actaattt	Homeobox
W-box promoter motif	8659751	8659756	ttgact	WRKY
W-box promoter motif	8660542	8660547	ttgacc	WRKY
W-box promoter motif	8661427	8661432	ttgact	WRKY
W-box promoter motif	8661636	8661641	ttgact	WRKY
W-box promoter motif	8659720	8659725	ttgacc	WRKY
ARF1 binding site motif	8660562	8660567	tgtctc	ARF
ATHB1 binding site motif	8659493	8659501	caataattg	HB
ATHB1 binding site motif	8659492	8659500	caattattg	HB
ATHB2 binding site motif	8660918	8660926	taatcatta	HB
ATHB5 binding site motif	8659493	8659501	caataattg	HB
ATHB5 binding site motif	8659492	8659500	caattattg	HB
ATHB6 binding site motif	8661889	8661897	caattatta	HB
CCA1 binding site motif	8660327	8660334	aaaaatct	MYB-related
DPBF1&2 binding site motif	8660091	8660097	acacgag	bZIP
MYB binding site promoter	8661918	8661925	aaccaaac	MYB
MYB4 binding site motif	8661051	8661057	aacaacc	MYB
MYB4 binding site motif	8661918	8661924	accaaac	MYB
RAV1-A binding site motif	8659791	8659795	caaca	ABI3VP1
RAV1-A binding site motif	8659918	8659922	caaca	ABI3VP1
RAV1-A binding site motif	8660442	8660446	caaca	ABI3VP1
RAV1-A binding site motif	8661325	8661329	caaca	ABI3VP1
RAV1-A binding site motif	8661414	8661418	caaca	ABI3VP1
RAV1-A binding site motif	8661640	8661644	caaca	ABI3VP1
RAV1-A binding site motif	8660804	8660808	caaca	ABI3VP1
RAV1-A binding site motif	8659938	8659942	caaca	ABI3VP1
RAV1-A binding site motif	8659929	8659933	caaca	ABI3VP1
RAV1-A binding site motif	8659447	8659451	caaca	ABI3VP1
RAV1-A binding site motif	8659419	8659423	caaca	ABI3VP1
LFY consensus binding site motif	8660895	8660900	ccactg	LFY
LFY consensus binding site motif	8661599	8661604	ccattg	LFY
ABRE-like binding site motif	8661342	8661349	tacgtgta	...
ARF binding site motif	8660562	8660567	tgtctc	...
BoxII promoter motif	8660579	8660584	ggttaa	...
BoxII promoter motif	8662158	8662163	ggttaa	...
GATA promoter motif [LRE]	8659340	8659345	agatag	...
GATA promoter motif [LRE]	8659423	8659428	tgataa	...
GATA promoter motif [LRE]	8659658	8659663	agataa	...
GATA promoter motif [LRE]	8659780	8659785	tgataa	...
GATA promoter motif [LRE]	8662121	8662126	tgataa	...
GATA promoter motif [LRE]	8659864	8659869	tgataa	...
GCC-box promoter motif	8662186	8662191	gccgcc	...
Ibox promoter motif	8659659	8659664	gataag	...
L1-box promoter motif	8660633	8660640	taaatgca	...
T-box promoter motif	8659382	8659387	actttg	...
T-box promoter motif	8659444	8659449	actttg	...
T-box promoter motif	8660597	8660602	actttg	...
T-box promoter motif	8661196	8661201	actttg	...
T-box promoter motif	8661096	8661101	actttg	...
SORLREP3	8660025	8660033	tgtatatat	...
SORLREP3	8660434	8660442	tgtatatat	...
SORLIP2	8660826	8660830	gggcc	...

(B)

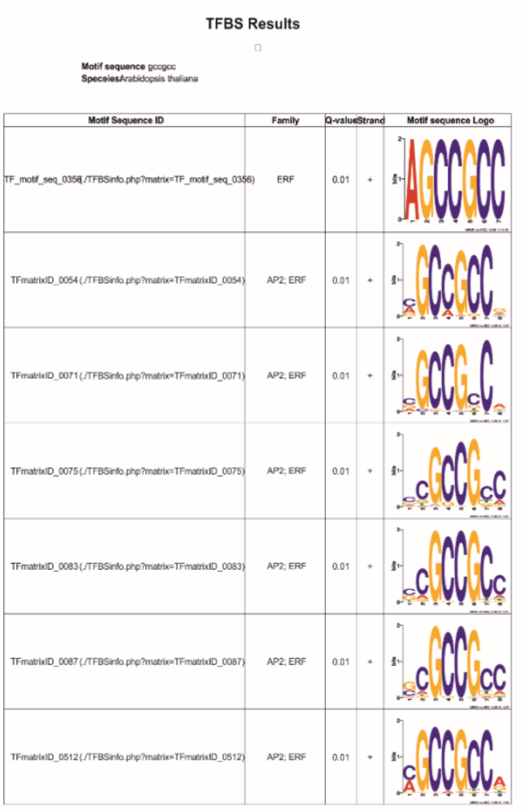

TFBS Results

Motif sequence gccgcc
SpeceiesArabidopsis thaliana

Motif Sequence ID	Family	Q-value	Strand	Motif sequence Logo
TF_motif_seq_0356(./TFBSinfo.php?matrix=TF_motif_seq_0356)	ERF	0.01	+	
TFmatrixID_0054 (./TFBSinfo.php?matrix=TFmatrixID_0054)	AP2; ERF	0.01	+	
TFmatrixID_0071 (./TFBSinfo.php?matrix=TFmatrixID_0071)	AP2; ERF	0.01	+	
TFmatrixID_0075 (./TFBSinfo.php?matrix=TFmatrixID_0075)	AP2; ERF	0.01	+	
TFmatrixID_0083 (./TFBSinfo.php?matrix=TFmatrixID_0083)	AP2; ERF	0.01	+	
TFmatrixID_0087 (./TFBSinfo.php?matrix=TFmatrixID_0087)	AP2; ERF	0.01	+	
TFmatrixID_0512 (./TFBSinfo.php?matrix=TFmatrixID_0512)	AP2; ERF	0.01	+	

Figure S2 – (A) *CIPK25* promoter analysis through AGRIScisDB platform (https://agris-knowledgebase.org/AtcisDB) highlighting various cis-elements in the 2 kb upstream of the gene coding sequence. **(B)** GCC-box targets found through PlantPAN 2.0 browser (http://plantpan2.itps.ncku.edu.tw/).

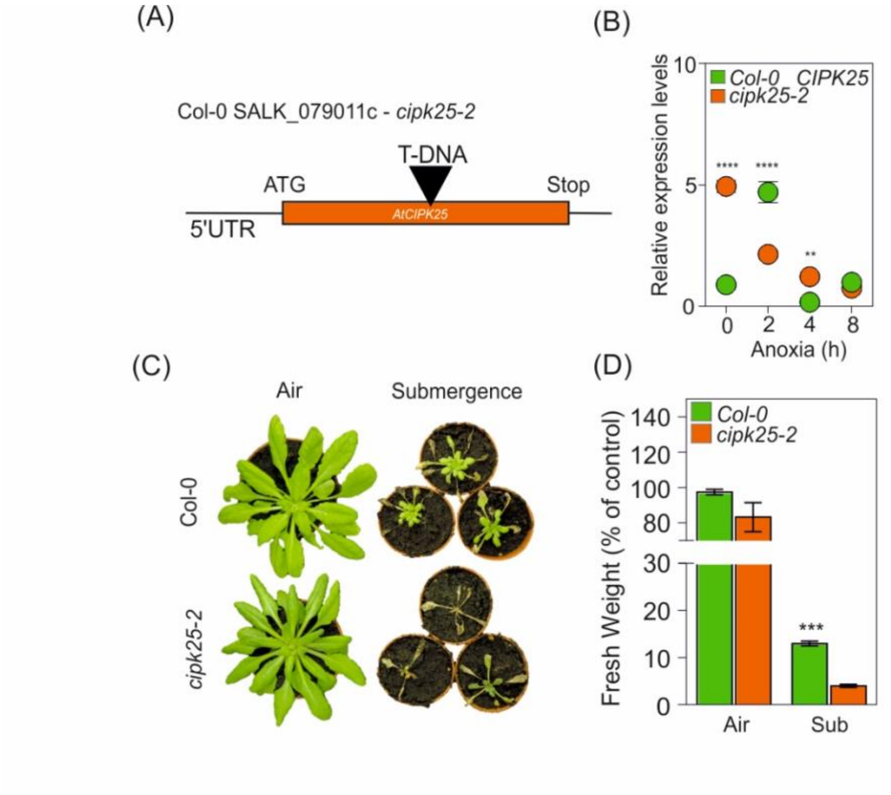

Figure S3 - (A) T-DNA insertion in the Arabidopsis mutant *cipk25-2* (background Col-0). **(B)** Gene expression analysis of *CIPK25* transcriptional level in Col-0 and *cipk25-2* mutant roots of 21-day-old plants grown on vertical plates. The value for Col-0 was arbitrarily set to one. Each value represents the mean ± SE (n=4). Statistical significance (*cipk25-2* vs. Col-0) was determined using Student's t-test, where **** $p<0.0001$, *** $p<0.001$, ** $p<0.01$. **(C, D)** Effect of 72 hours of dark submergence on the survival of the *cipk25-2* Arabidopsis mutant, where % represent the mean ± SE of shoot fresh weight measurements in comparison to control (n=3). Statistical significance (*cipk25-2* vs. Col-0) was determined using Student's t-test, where *** $p<0.001$.

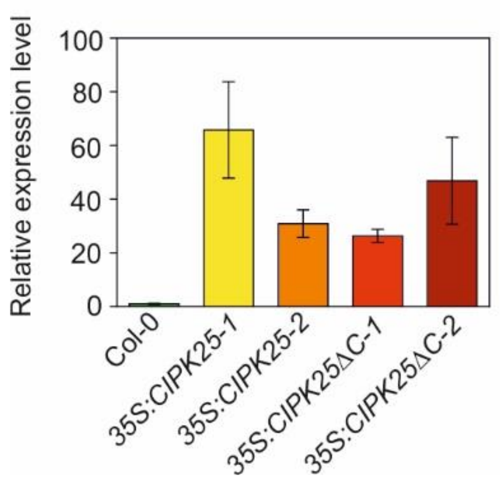

Figure S4 – Relative expression level of *CIPK25* gene in 35S overexpressing transgenic lines. Data are the mean of two biological replicates of four days seedlings ± SD.

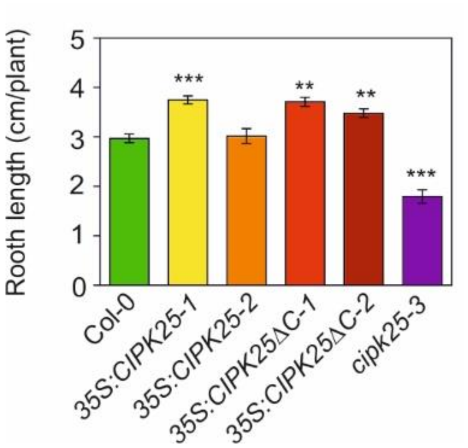

Figure S5 - Root length of seedlings after 14 days of growth on plates in air. Each value represents the mean ±SE (98<n<104). Statistical significance (Col-0 vs. other genotypes) was determined using Student's t-test, where ** $p<0.01$, *** $p<0.001$.

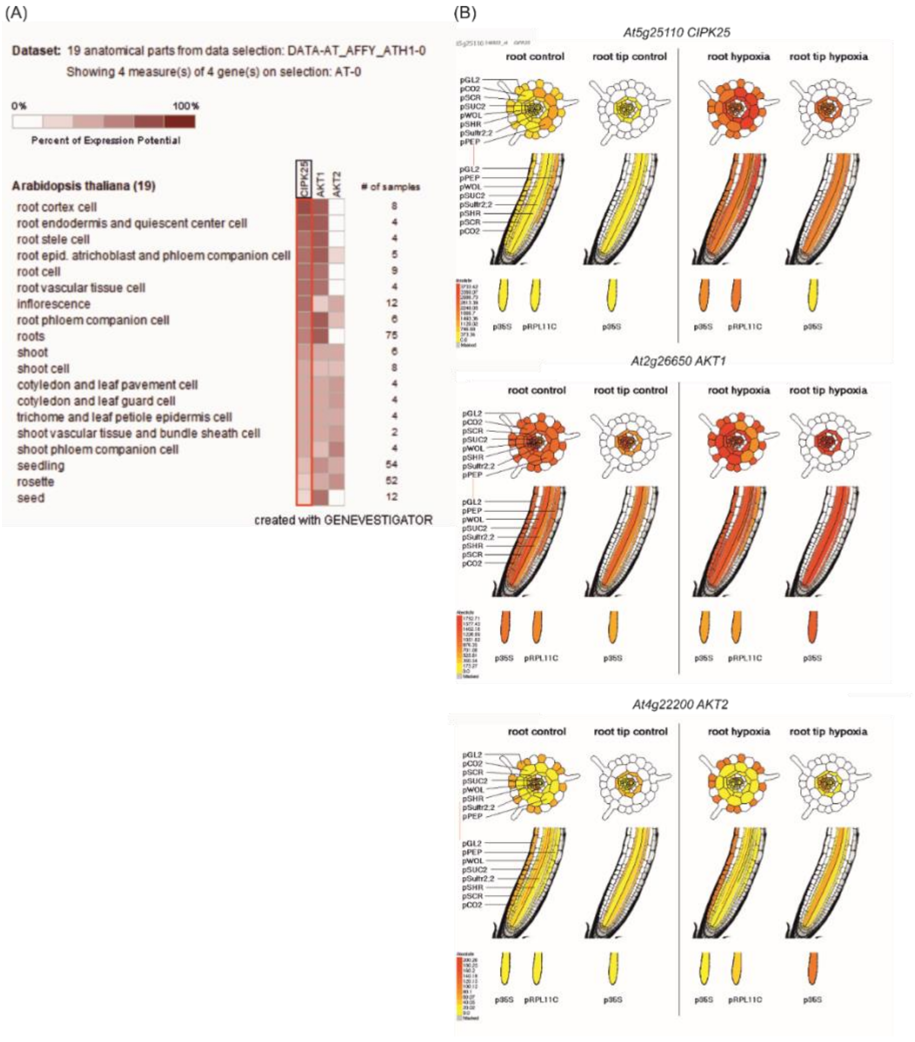

Figure S6 – (A) Comparison between *CIPK25*, *AKT1 and AKT2* expression pattern under O_2 shortage in different Arabidopsis tissues using Genevestigator software (https://genevestigator.com/gv/). **(B)** Absolute level of cell type-specific expression of *CIPK25*, *AKT1* and *AKT2* genes in roots under aerobic and hypoxic conditions using Arabidopsis Translatome eFP browser (http://efp.ucr.edu/).

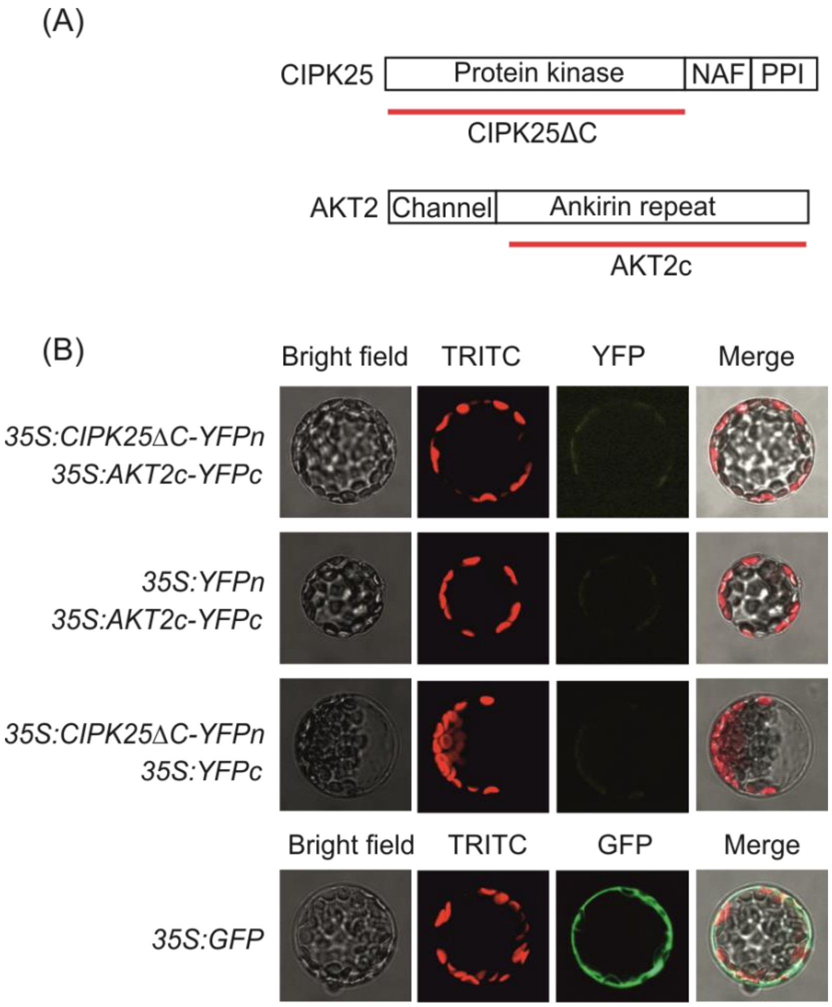

Figure S7 – **(A)** Constructs used for bimolecular fluorescence complementation. **(B)** Bimolecular fluorescence complementation (BiFC) assay for interaction between CIPK25ΔC and AKT2 C-terminus. Alternate empty vectors were used as negative controls. pAVA vector was used as 35S:GFP positive control of transformation. The experiment was run in triplicate.

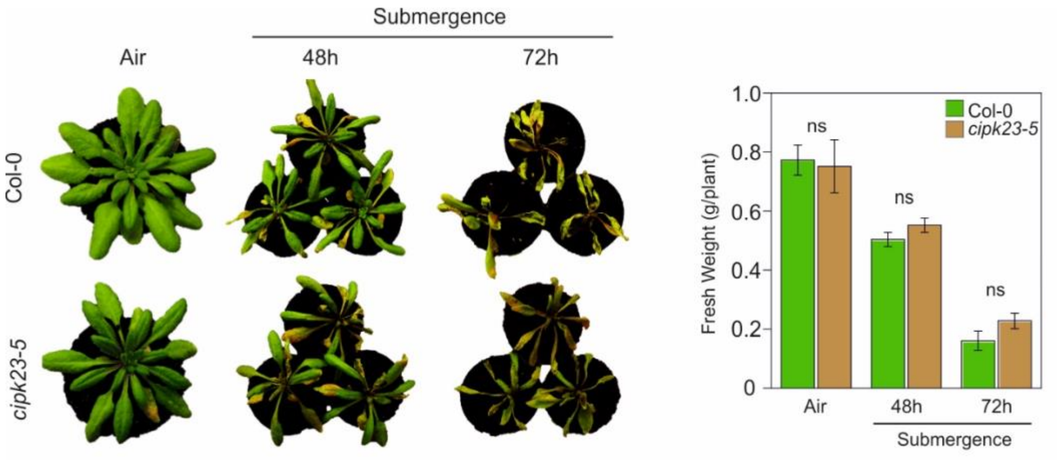

Figure S8 – **(A)** Effect of 48 and 72 hours of dark submergence on the survival of the cipk23-5 Arabidopsis mutant, where graph **(B)** represent the mean ± SE (n=3) of shoot fresh weight measurements in comparison to control. Statistical significance (*cipk23-5* vs. Col-0) was determined using Student's t-test.

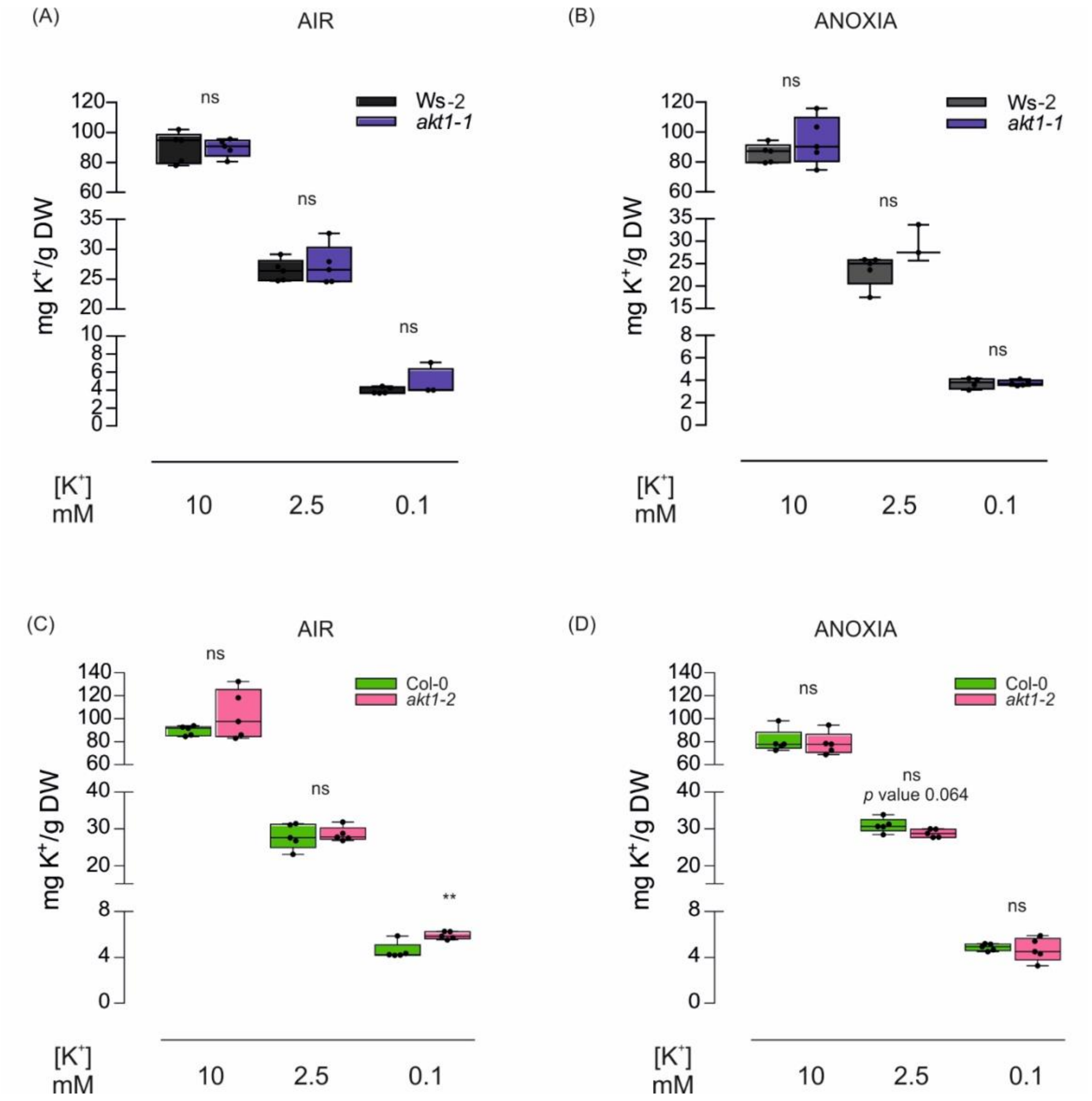

Figure S9 – Potassium concentration of Ws-2 and *akt1-1* mutant seedlings grown under different K+ concentrations in air (**A**) and anoxia (**B**) with relative box plot graphs (mean ± SD, n=5). Potassium concentration of Col-0 and *akt1-2* mutant seedlings grown under different K+ concentrations in air (**C**) and anoxia (**D**) with relative box plot graphs (mean ± SD, n=5). Statistical significance (*akt1-1* vs. Ws-2 and *akt1-2* vs. Col-0) was determined using Student's t-test.
